# Comparative Cerebroprotective Potential of d- and l-Carnosine Following Ischemic Stroke in Mice

**DOI:** 10.3390/ijms21093053

**Published:** 2020-04-26

**Authors:** Saurabh Jain, Eun-Sun Kim, Donghyun Kim, David Burrows, Milena De Felice, Minyeong Kim, Seung-Hoon Baek, Ali Ali, Jessica Redgrave, Thorsten R. Doeppner, Iain Gardner, Ok-Nam Bae, Arshad Majid

**Affiliations:** 1 Department of Neuroscience, SITraN, University of Sheffield, Sheffield S10 2HQ, UK; 2 College of Pharmacy, Institute of Pharmaceutical Science and Technology, Hanyang University, Ansan 15588, Korea; 3 College of Pharmacy and Research, Institute of Pharmaceutical Science and Technology (RIPST), Ajou University, Suwon 16499, Korea; 4 Department of Neurology, University Medical Center Goettingen, 37075 Goettingen, Germany; 5 Translation DMPK Sciences, Simcyp Division, Certara, Sheffield S1 2BJ, UK

**Keywords:** stroke, d- and l-carnosine, efficacy, pharmacokinetics, MCAO, neuroprotection

## Abstract

l-carnosine is an attractive therapeutic agent for acute ischemic stroke based on its robust preclinical cerebroprotective properties and wide therapeutic time window. However, large doses are needed for efficacy because carnosine is rapidly degraded in serum by carnosinases. The need for large doses could be particularly problematic when translating to human studies, as humans have much higher levels of serum carnosinases. We hypothesized that d-carnosine, which is not a substrate for carnosinases, may have a better pharmacological profile and may be more efficacious at lower doses than l-carnosine. To test our hypothesis, we explored the comparative pharmacokinetics and neuroprotective properties of d- and L-carnosine in acute ischaemic stroke in mice. We initially investigated the pharmacokinetics of d- and L-carnosine in serum and brain after intravenous (IV) injection in mice. We then investigated the comparative efficacy of d- and l-carnosine in a mouse model of transient focal cerebral ischemia followed by in vitro testing against excitotoxicity and free radical generation using primary neuronal cultures. The pharmacokinetics of d- and l-carnosine were similar in serum and brain after IV injection in mice. Both d- and l-carnosine exhibited similar efficacy against mouse focal cerebral ischemia. In vitro studies in neurons showed protection against excitotoxicity and the accumulation of free radicals. d- and l-carnosine exhibit similar pharmacokinetics and have similar efficacy against experimental stroke in mice. Since humans have far higher levels of carnosinases, d-carnosine may have more favorable pharmacokinetics in future human studies.

## 1. Introduction

Stroke is the second leading cause of death worldwide, accounting for 11.13% deaths each year [1]. Thrombectomy and thrombolysis with recombinant tissue plasminogen activator (rtPA) are the only approved acute therapies. However, both therapies have short therapeutic time windows, which, in most cases, is 4.5–6 hrs. There is, therefore, a desperate need for new therapies that are safe, effective and have a longer therapeutic time window.

Despite many promising preclinical studies, none of the experimental drugs has succeeded in human clinical trials. These drugs include glutamate antagonists, anti-inflammatory agents, ion channel modulators, free radical scavengers, γ-Aminobutyric acid receptor antagonists, serotonin agonists and caspase inhibitors [2,3,4]. The reasons why these drugs failed are not clear, but may include low efficacy, intolerable side effects and the short therapeutic time windows of these compounds [5,6,7,8,9].

l-carnosine (β-alanyl-l-histidine) is an endogenous dipeptide which is expressed in many tissues of the body including the brain and blood [10]. It exhibits pleiotropic biological activities such as heavy metal chelation [11,12,13,14], scavenging of reactive oxygen species (ROS) and reactive nitrogen species (RNS) [15,16], cytosolic hydrogen ion buffering [17] and anti-excitotoxicity [18,19]. Moreover, it is a powerful inhibitor of matrix metalloproteinase 9 [13].

It is well established that cerebral damage caused by ischemic strokes is mediated through excitotoxicity, caused by stroke-induced high glutamate levels [20]. This mechanism can be modelled in vitro by exposing primary cortical neurons to NMDA (a glutamate agonist) or depriving the cells of oxygen and glucose for a specified period. Several studies have shown that l-carnosine is protective in primary mouse neuronal and astrocytic cultures against NMDA induced excitotoxicity and oxygen-glucose deprivation. l-Carnosine also improves histological and neurological outcomes in temporary and permanent occlusion models in rodents [11,13,14,21] which are used to mimic human stroke.

However, in humans, l-carnosine has a short half-life due to its rapid inactivation by serum and tissue carnosinases [22], which might be a hurdle for its clinical application. To date, two carnosinases, CN1 and CN2, that are encoded by CNDP1 and 2, respectively, have been identified. CN1 is specifically expressed in serum and brain tissue and is identified as human serum carnosinase [23]. Allelic variations of the serum carnosinase CNDP1 gene, which result in reduced enzyme levels in humans, have been shown to be associated with protection against diabetic nephropathy [24]. CN2, on the other hand, is cytosolic and has broad activity against various peptides [23].

d-carnosine is a nonnatural isomer of l-carnosine and has the same crystalline structure and melting point. The carnosinase-resistant compound d-carnosine appears to be as effective as l-carnosine in preventing renal disease and metabolic dysfunction in Zucker rats [25]. d-carnosine is less sensitive to hydrolysis by carnosinases, is stable in human plasma and is able to cross the blood brain barrier [26]. Due to its resistance to carnosinases, it is hypothesized that it will have a more favorable pharmacokinetic profile in humans. However, the efficacy of d-carnosine in models of cerebral ischemia is unknown and needs to be determined. Herein, we compared the pharmacokinetic properties of l- and d-carnosine, as well as the comparative efficacy of these enantiomers in in vitro models of cerebral ischemia and in a mouse model of focal ischemia.

## 2. Results

### 2.1. l- and d-Carnosine in Serum and Brain Levels Postintravenous Administration

**Figure 1:**
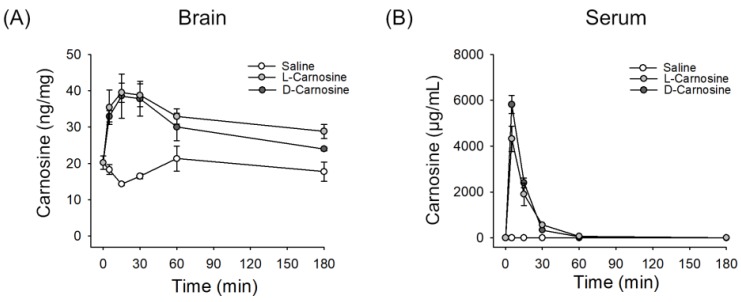
Concentration-time curves of carnosine in brain and serum via intravenous administration in healthy mice; D-carnosine (*n* = 4), L-carnosine (*n* = 5) and saline (*n* = 5). Saline was used as vehicle throughout the study. (**A**) Levels of carnosine measured in brain at different time points (0 to 180 min). (**B**) Levels of carnosine measured in serum at different time points (0 to 180 min). Mean ± SEM.

After a single intravenous injection of saline or L-carnosine (1000 mg/kg) or d-carnosine (1000 mg/kg) to mice, serum or brain samples were isolated and the levels of carnosine were measured by LC-MS/MS. Basal level of carnosine in mouse brain was ~ 20 ng/mg tissue (i.e., ~ 88.4 μmole/kg), which is similar to previous reports [10,27]. l- and d-carnosine levels were plotted at different time points (5, 15, 30, 60, 180 and 360 min). Based on these data, pharmacokinetic parameters were determined and are shown in Table 1 and Figure 1. The AUC (area under curve), Cmax (peak serum concentration) and T1/2 (half-life) and CL were similar between l- and d-carnosine. The Vss (steady state volume of distribution), however, was higher for l-carnosine.

### 2.2. Both d- and l-Carnosine Exhibit Protection against Mouse Transient Focal Ischemia

**Figure 2:**
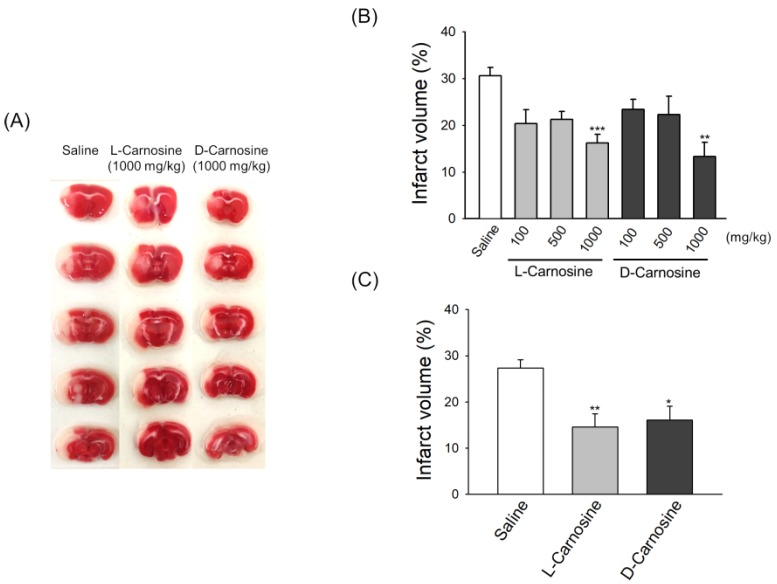
Neuroprotective effects of l- and d-carnosine against ischemic damage in transient focal ischemic mouse model. (**A** and **B**) Representative images of TTC staining of mouse brain (**A**) and infarct volumes (**B**) after 48 h postintraperitoneal administration of saline, d- or l-carnosine (100 mg/kg (*n* = 6), 500 mg/kg (*n* = 6) or 1000 mg/kg (*n* = 6)) at onset of reperfusion. Mean ± SEM. ** *p* < 0.01, and *** *p* < 0.001 vs saline (*n* = 7). (**C**) Comparison of infarct volume between intravenously administered saline (*n* = 10), l-carnosine (*n* = 12; 1000 mg/kg) or d-carnosine (*n* = 13; 1000 mg/kg) when delivered at 2 h postischemia. Mean ± SEM. * *p* < 0.05, and ** *p* < 0.01.

To determine the relative cerebroprotective potential of D- and L-carnosine in transient focal ischemic damage, MCAO occlusion was induced for 60 min. The mortality rate throughout our experiments was less than 10%. We initially tested the relative efficacy of different doses of l- and d-carnosine (Figure 2A,B) when administered IP at the onset of reperfusion. A significant reduction in the infarct volume was detected by triphenyl tetrazolium chloride (TTC) staining of brain slices obtained after 48 h post-t-MCAO (Figure 2A). In the case of l-carnosine, the infarct volume was significantly reduced by 47.4% (*p* = 0.0045), 30.94% and 33.4% in comparison to saline when the drug was delivered at 1000, 500 and 100 mg/kg respectively. Similarly, in the d-carnosine group, infarct volume was reduced by 57.2% (*p* = 0.0004), 27.8% and 23.6% at delivery doses of 1000, 500 and 100 mg/kg respectively.

We also tested the efficacy of both L- and D-carnosine when administered intravenously 2 h post-t-MCAO at 1000 mg/kg (Figure 2C). Mice were sacrificed 48 h post-MCAO to assess the extent of infarction. As shown in Figure 2C, both l- and d-carnosine treatment significantly reduced infarct volume when delivered at 1000 mg/kg in mice by 53.8% (*p* = 0.008) and 52.1% (*p* = 0.01), respectively. Figure 2A is a representative image of TTC stained brain slices obtained after 48 h post-t-MCAO showing infarct in saline and drug treated mice.

### 2.3. Effect of l- and d-Carnosine on ROS Accumulation in Primary Neurons

**Figure 3:**
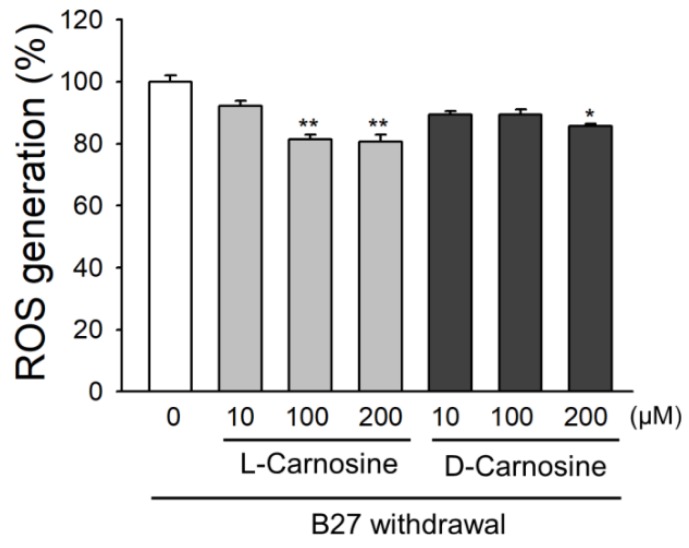
L- and D-carnosine reduce ROS accumulation in primary mouse neurons following 24 h B27 withdrawal. Neurons were loaded with H2DCFDA (20 µM) and oxidative stress induced by the removal of B27 supplement. Values expressed as a percentage relative to control condition (no carnosine). *n* = 3 experiments. Mean ± SEM. * *p* < 0.05, and ** *p* < 0.01.

To further elucidate the mechanism for the neuroprotective effects of l- and d-carnosine, we examined whether the two enantiomers of carnosine affect oxidative stress. Oxidative stress arises from an imbalance between ROS production and removal. Withdrawal of B27 supplement has been successfully used as an in-vitro model to induce oxidative stress in primary neurons. Both l- and d-carnosine reduced ROS accumulation when delivered at different doses during oxidative stress. ROS production was measured using H2DCFDA, which mainly reacts with superoxide anions, hydroxyl radicals and hydrogen peroxide. Withdrawal of B27 caused a significant increase in DCF fluorescence, which is attenuated by l- and d-carnosine. As shown in Figure 3, a significant reduction in ROS accumulation was achieved in the presence of 100 µM or 200 µM of l-carnosine. However, d-carnosine was only found to be effective at a dose of 200 µM. L-carnosine attenuated the ROS accumulation by 18.6% and 19.3% at a dose of 100 µM (*p* = 0.0032) or 200 µM (*p* = 0.0021), respectively, while d-carnosine reduced oxidative stress by 14.5% when delivered at 200 µM (*p* = 0.0438).

### 2.4. Neuroprotection in Primary Cortical Neuronal Cultures

**Figure 4:**
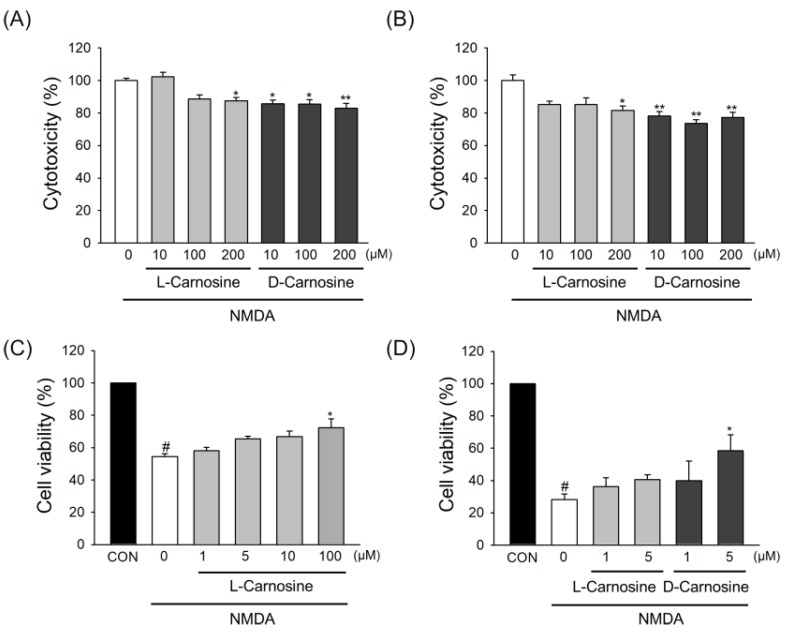
l- and d-carnosine reduce NMDA-induced excitotoxicity in primary mouse and rat neurons. (A and B) Primary neurons isolated from mice (**A**) or rats (**B**) were pretreated with l- or d-carnosine for 24 h prior to NMDA stimulation as described in Method. Wells were washed and original media replaced, in the presence or absence of L- or D-carnosine. Cytotoxicity was measured at 24 h by LDH release. (**C**,**D**) Cell viability was measured by MTT assay in primary mouse neurons treated with NMDA in the presence or absence of L- or D-carnosine. Concentration-dependent protective effect of L-carnosine (**C**) or comparative effect of L- or D-carnosine (**D**) was observed. Values expressed as a percentage relative to control condition (no carnosine). **A**, *n* = 3; **B**, *n* = 4; **C**, *n* = 3; **D**, *n* = 3 experiments. Mean ± SEM. # *p* < 0.05 vs. control cells without NMDA treatment; * *p* < 0.05, and ** *p* < 0.01 vs. NMDA-treated cells.

Only cultures which were more than 90% positive for specific neuronal marker MAP2 were used for NMDA induced excitotoxicity. We examined the neuroprotective potential of l- and d-carnosine in NMDA exposed mouse and rat cortical neurons. As shown in Figure 4A, l-carnosine elicited neuroprotection at 200 µM, whereas, d-carnosine elicited neuroprotection when used at a dose of 10, 100 and 200 µM. l-carnosine did not show neuroprotection when tested at 10 and 100 µM; however, it reduced the cell death by 12.2% (*p* = 0.0307) at 200 µM when assessed using LDH assay. Interestingly, d-carnosine elicited neuroprotection against NMDA excitotoxicity when tested at 10, 100 and 200 µM, and reduced cell death by 13.1% (*p* = 0.0183), 13.3% (*p* = 0.0164) and 15% (*p* = 0.0058), respectively in mouse cortical neurons.

Similarly, when carnosine enantiomers were tested in rat neurons (Figure 4B), l-carnosine significantly reduced cell death by 18.5% (*p* = 0.0201) at a dose of 200 µM. On the other hand, in rat neurons, d-carnosine reduced the cell death by 21.9% (*p* = 0.0061), 26.4% (*p* = 0.0013) and 22.8% (*p* = 0.0044) when dosed at 10, 100 and 200 µM, respectively.

To measure cell viability based on cell metabolism capacity, primary neurons isolated from mouse were treated with NMDA, and an MTT assay was conducted. Cells were pretreated with l- or d-carnosine for 24 h prior to NMDA stimulation and cells were maintained for an additional 24 h after NMDA treatment in the absence or presence of l- or d-carnosine. While NMDA significantly reduced cell viability, both L-carnosine and D-carnosine showed significant protection against decrease of cell viability by NDMA (Figure 4C,D).

## 3. Discussion

Despite the availability of thrombolytic therapy and, more recently, thrombectomy, there is still an urgent need for new acute stroke therapies. Based on preclinical studies, there has been significant interest in developing carnosine as a therapeutic agent in many diseases including stroke [28]. We and other groups have shown that l-carnosine has robust cerebro-protective properties, even when administered up to 9 h after the onset of experimental stroke [10]. However, human translation of these studies to clinical trials in stroke has been complicated by the rapid breakdown of l-carnosine in humans by carnosinases CN1 and CN2 [25], which are not present in abundance in rodents.

CNDP1 encodes the secreted serum carnosinase with high specificity for l-carnosine, whereas CNDP2 encodes tissue or cytosolic carnosinase, which has less specificity and is a general dipeptidase [29]. Sequence alignments of human CNDP1 and CNDP2 with mouse CNDP2 show a homology of 53 and 91%, respectively [30]. Both these enzymes possess the ability to hydrolyze dipeptides, including l-carnosine [31]. The expression pattern of CNDP1 varies in humans and rodents. Human CNDP1 is expressed extensively in the brain and liver, whereas in rodents, CNDP1 is mainly expressed in the kidney [32]. The d- enantiomer of carnosine is not thought to be a substrate for serum carnosinases, and thus, may be a superior compound for testing in human studies. d-carnosine is also able to cross the BBB and maintain the same activity of l-carnosine in- vitro [26]. Due to this relative resistant to degradation by carnosinases [25], we investigated the relative neuroprotective potential of d-carnosine versus l-carnosine in vitro and in a mouse model of experimental stroke.

Against our initial hypothesis, our study showed that both d- and l-carnosine had similar pharmacokinetic parameters. Similar pharmacokinetics allowed a direct comparison to be made of efficacy in mice. The concentrations achieved in the brain for the two enantiomers showed no statistical difference at any of the time points, suggesting that the concentrations of the two compounds in the brain were the same for the duration of the experiment. The plasma concentrations showed some differences that reached statistical significance for the 5, 30 and 180 min time points. There were, however, some limitations in our pharmacokinetic work. Although the measurements of d-and l-carnosine in the plasma and brain were made at different time points, each animal in the study provided a single plasma and brain sample, making it difficult to determine the pharmacokinetic parameters in each animal. Using a naïve pooled and sparse sampling approach [33,34] and combining all the available data in a single analysis, the plasma pharmacokinetic parameters were determined (Table 1). Variability cannot be assessed with naïve pooled approaches [34]. The results of this analysis suggested that the volume of distribution at steady state of l-Carnosine was bigger than that of d-carnosine, whilst the area under the plasma concentration time curve and the clearance were similar for the two compounds. The reasons for this difference in apparent volume of distribution at steady state are not clear, and could be assessed in a dedicated future pharmacokinetic study that would allow a more comprehensive comparison to be made of the two compounds. Even though d- Carnosine is not thought to be a substrate for serum carnosinases, it is possible that degradation occurred by other nonspecific dipeptidases in the serum [23].

We administered the drug intravenously because in a future clinical trial, carnosine would be administered via the same route. Our data show that both d-and l-Carnosine are highly efficacious in protecting against brain damage when administered intravenously, and are safe and well tolerated at doses up to 1000 mg/kg in mice. Both agents exhibited robust neuroprotection when administered at reperfusion or 2 h after reperfusion. Similarly, both agents exhibited efficacy against NMDA excitotoxicity in mouse and rat cortical neurons. In fact, d-carnosine elicited greater efficacy in comparison to l-carnosine against excitotoxicity in mouse and rat cortical neurons. Irrespective of the mechanism involved in triggering ischemic stroke, a cascade of events leads to an increase in ROS production; for this reason, we tested the ability of l and d-carnosine against free radical generation accumulation in neurons by nutrient withdrawal. Both carnosine enantiomers reduced ROS accumulation, which, in turn, could affect a cascade of events that can lead to neuroprotection. However, we acknowledge that the effect on ROS production is relatively small, and other pathways are involved in the neuroprotection that was observed.

Although the aim of this study was to explore the comparative efficacy of d- and l-carnosine after IV administration in experimental stroke, d-carnosine has been reported to be less well transported by PEPT1/2, and the synthetic octyl-d-carnosine ester has been proposed as a better version of d-carnosine [35]. Previous studies have also explored natural and nonnatural alternatives of carnosine including carnosinol [36] and l-anserine [37,38]. These agents may also have therapeutic utility in stroke, and may warrant further investigation.

The upper concentration of d- or l-carnosine tested in our in vitro experiments was 200 µM. The concentration ranges tested in previous in vitro studies vary from nanomolar to millimolar levels [39,40,41]. The therapeutic dose of carnosine against rodent ischemic stroke models was 1000 mg/kg, and micromolar concentrations would be achievable in vivo. In rats, serum levels of carnosine were 15~20 mmol/L at 15 min after intravenous injection of l-carnosine 2000 mg/kg [42]. In the current study, the serum levels of d- or l-carnosine (1000 mg/kg) were 5818 μg/mL and 4011 μg/mL (Table 1), which correspond 25.7 mmol/L and 17.7 mmol/L, respectively, in mice at 5 min after injection. The question of whether higher serum concentrations of d-carnosine are achievable in humans with lower treatment doses needs further study.

There are several limitations of our study. We only tested short-term histological outcomes using TTC; comparative efficacy on long-term histological and functional outcomes would have been useful. In addition, a comparison of the therapeutic time window was not done. Future in vitro studies on the neuroprotective mechanisms of d-carnosine will be required to fully explain its in vivo efficacy. Although the antioxidant and antiexcitotoxic activities of d-carnosine were statistically significant and were comparable to those of L-carnosine in isolated neurons, these effects may not fully explain the in vivo efficacy. It is likely that these effects contribute simultaneously to other beneficial effects that result in protection against ischemic damage, such as protective effects on ischemic autophagy or the reduction of matrix metalloproteinase activation that have been demonstrated with l-carnosine [11,21]. Further studies using other models, such as oxygen glucose deprivation models, would be useful to further explore the mechanisms of action of d-Carnosine. Despite these limitations, we believe that our study adds useful new insights to the body of literature on carnosine.

In summary, our work, shows, for the first time, that d-carnosine exhibits robust cerebroprotective activity in acute ischemic stroke, and may be an attractive alternative to l-carnosine for human clinical trials.

## 4. Materials and Methods

### 4.1. Animals

Six- to eight-week-old male C57bl/6J (20–25 g) mice were purchased from Charles River, UK for use throughout this study. The use of animals and aseptic surgical procedures were in accordance with the guidelines stated under a license obtained from the Home Office subject to the Animals (Scientific Procedures; Act, 1986; license approval code: 70/8408; date: 16 February 2015). Mice were housed at 22 °C under a 12 h light–dark cycle and were fed a commercial diet. The mice were allowed to acclimatize to new conditions for one week upon arrival before experimental use. For each study, mice were divided into 3 groups: A control group (saline), l-carnosine and d-carnosine-treated group. All studies were randomized and carried out in a blinded manner both for allocation to treatment and assessment of outcomes.

### 4.2. Isolation of Serum and Brain Samples for Kinetic Studies of l- or d-Carnosine in Mice

Mice were randomly divided into the treatment groups (*n* = 4~5 mice/group for each time point) and given a single intravenous injection of saline or l-carnosine (1000 mg/kg) or d-carnosine (1000 mg/kg) through the tail vein. Anesthesia was induced by isoflurane inhalation and maintained during intravenous injection. Blood samples were collected by cardiac puncture at 0, 5, 15, 30, 60, 180 and 360 min postadministration of carnosine. Blood was incubated at room temperature for 30 min to allow clotting to occur, followed by centrifugation at 12,000 g to isolate serum. Remaining blood was removed from the body through perfusion with phosphate-buffered saline, and the brain was carefully isolated. Aliquots of brain tissue (300 mg) and serum (150 μL) were stored at −70 °C and used for analysis.

### 4.3. Determination of Carnosine Levels in Serum or Brain Using LC-MS/MS

Carnosine levels in serum or brain were determined using liquid chromatography tandem-mass spectrometry (LC-MS/MS) as previously described [43,44] with a slight modification. H-Tyr-His-OH (Bachem AG, Köln, Germany) was used as internal standard (IS). The entire procedure for sample and standard preparation was carried out on ice. Serum (150 μL) or homogenized brain tissue (300 mg) were deproteinized using an ultrasonicator (VCX 500, Sonics & Materials Inc., Newtown, CT, USA) with 1 mL of 1 M TCA and 50 μL of 3 M TCA, respectively. After spiking the IS (10 μg for brain and 200 ng for serum), each homogenate was centrifuged at 9000 g for 15 min at 4 °C. The supernatant was used for the assay of carnosine concentrations. Calibration curves were obtained by spiking known concentrations of l- or d-carnosine (Sigma-Aldrich, St. Louis, MO, USA) into homogenates of brain or serum obtained from untreated mice, and samples were processed as described above.

Chromatographic separation and quantitation were performed using an LC-MS/MS system composed of HPLC (Ultimate 3000, Dionex, Rome, Italy) coupled with electrospray ionization (ESI) and triple quadrupole-ion trap mass spectrometry (3200 Qtrap, AB Sciex, Ontario, Canada). Synergi C18-L (Phenomenex 150 × 4.6 mm, 4 μm particle size) column was used with a 0.8 mL/min flow rate of mobile phase. Isocratic elution of 75/25 (5 mM heptafluorobutyric acid/acetonitrile) was used for brain samples and gradient elution (95/5 to 80/20 for 6 min and holding for 4 min) was used for serum samples. The injection volume was 5 μL. For ESI source, ion spray voltage was 4.5 kV and capillary temperature was set at 650 °C which provided optimum ionization of each analyte. A multiple reaction monitoring (MRM) in positive mode was used to detect carnosine (m/z 227 > 110 as quant ion, 156 and 83 as qualifier), histidine (m/z 156 > 110 as quant ion, 83 and 82 as qualifier) and IS (m/z 319 > 110 as quant ion, 156 and 91 as qualifier). Pharmacokinetic parameters were calculated by noncompartmental analysis using a sparse sampling and naïve pooled approach (WinNonLin (Phoenix 64 Build 8.0.0.3176), Certara, Mountain View, CA, USA) [33,34].

### 4.4. Transient Focal Cerebral Ischemia

Mice were anesthetized by inhalation of 5% isoflurane (in 100% Oxygen) and maintained at 1.5% isoflurane throughout the surgical procedure. Body temperature was monitored throughout surgery using a rectal probe and maintained at 37 + 0.5 °C using a heating pad (World Precision Instruments, Hitchin, Hertfordshire, UK). Laser Doppler flowmetry (Moor Instruments, Sussex, UK) was used to monitor cerebral blood flow. Focal cerebral ischemia was induced by middle cerebral artery occlusion (MCAO) by insertion of an intraluminal monofilament. Briefly, a small incision was made in the skin overlying the temporalis muscle and a 0.7 mm flexible optical laser Doppler probe was positioned on the superior part of the temporal bone (6 mm lateral and 2 mm posterior from bregma), secured by superglue (Loctite 454). A midline incision was made on the ventral side of the neck and the left common carotid artery (CCA) was isolated and ligated. Another ligature was tied on the left external carotid artery (ECA) and a loose knot was tied onto the internal carotid artery (ICA) as well as CCA just below the bifurcation. A very fine incision was made on the lower end of CCA and the monofilament (Doccol Corporation, Sharon, MA, USA) was advanced approximately 9 mm distal to the bifurcation into the ICA until it reached the distal end of middle cerebral artery (MCA). Relative cerebral blood flow was monitored for initial 10 min postfilament insertion to confirm at least 70% reduction of preischemic values. The animals were kept under anesthesia for the ischemic period of 60 min, after which the filament was withdrawn and reperfusion (confirmed using LD) was allowed to take place. The knots were untied and the animals were allowed to recover at 37 °C for 1 h before they were transferred to their normal cages.

### 4.5. Drug Treatment in the MCAO Model

In the first set of experiments, 60 min transient MCAO (t-MCAO) was induced in mice weighing 20–25 g. At the onset of reperfusion, saline or different doses of drugs (l- and d-carnosine; 100, 500 or 1000 mg/kg) were delivered intraperitoneally (IP). Postrecovery, the animals were allowed to recover, placed back in their normal environment for 48 h and then euthanized. (IP administration was used as it was technically difficult to do IV injections at reperfusion). In a second set of experiments, drugs and saline were administered intravenously 2 h post-t-MCAO at 1000 mg/kg.

### 4.6. 2, 3, 5-Triphenyltetrazolium Chloride (TTC) Staining

Forty-eight hours post-t-MCAO, mice were deeply anaesthetized with 5% isoflurane, brains were removed and coronal slices with a thickness of 1 mm were prepared. Brain slices were immersed in 2% TTC (Sigma Aldrich) solution and incubated at 37 °C for 20 min. The area of infarction was traced and measured using image J analysis software. The infarct area was also corrected for edema: (1-(total ipsilateral hemisphere-infarct region)/total contralateral hemisphere) × 100. Total infarct volume was calculated as the sum of all infarct areas multiplied by each section thickness.

### 4.7. In Vitro Culture of Primary Cortical Neurons

Primary cortical neurons were prepared from embryonic day 16 C57bl/6J mouse pups or embryonic day 18 Wistar rat pups. Briefly, cerebral cortices were physically and chemically dissociated with 0.25% trypsin-EDTA (Gibco, Waltham, MA, USA) in dissociation medium (HBSS) at 37 °C for 15 min followed by trituration. The dissociated cells were checked under a microscope for single cells and plated out on poly-l-lysine (Sigma-Aldrich) -coated, 24-well plates (187,500 cells/well) for the excitotoxicity assays, and 96-well plates (47,000 cells/well) for the oxidative stress assays. Neuronal cultures were maintained in B27 neurobasal medium with Glutamax and pen-strep (Gibco) in a 5% CO_2_ incubator at 37 °C, and cells were used at 11–14 days in vitro for experiments.

### 4.8. Oxidative Stress Assay

Cytosolic oxidative stress was determined using dichlorofluorescein (DCF) fluorescence. Primary neurons were loaded with 20 µM 2′, 7′-dichlorodihydrofluorescein diacetate (H2DCFDA; Invitrogen) for 45 min, washed and oxidative stress was induced by B27-withdrawal for 24 h, in the presence or absence of l- or d-carnosine. The fluorescence of oxidized DCF was read after 24 h at Ex485 nm/Em530 nm using the Pherastar FS platereader.

### 4.9. Excitotoxicity Assay

Neurons were pretreated with l- or d-carnosine for 24 h prior to NMDA stimulation (30 µM) for 30 min. Post-NMDA stimulation, the cells were washed and incubated back in the original medium in the presence or absence of l- and d-carnosine. Wells were washed and the B27-neurobasal medium was replaced in the presence or absence of L- or D-carnosine. Cytotoxicity was measured after 24 h using the Pierce cytotoxicity lactate dehydrogenase (LDH) assay kit. Cell viability was measured by 3-[4–Dimethylthiazol-2-yl]-2,5-diphenyltetrazolium bromide (MTT) assay, which reflects cell metabolism [45,46]. After NMDA removal, the cells were evaluated for cell viability after 24 h with the addition of fresh media containing each drug. MTT (Sigma Aldrich; final 5 mg/mL) was added to each well and the cells were incubated for 2 h at 37 °C in the dark. Insoluble formazan was dissolved by the addition of 100 μL of DMSO and the absorbance (570 nm) was measured using a multimode plate reader (EnSpire, PerkinElmer, Waltham, MA, USA).

### 4.10. Statistical Analysis

All values are presented as means ± standard error of means (SEM) unless otherwise stated. Subgroup comparisons were analyzed using one-way analysis of variance (ANOVA), followed by Tukey′s or Dunnett’s multiple-comparison test. The Prism analysis software was used to perform all statistical analysis; *p* < 0.05 was considered statistically significant.

## Figures and Tables

**Table 1:** Pharmacokinetic analysis of D- and L-carnosine in serum (*n* = 4~5).

	Half-Life (min)	Cmax (5 min) (μg/mL)	AUC (μg/mL min)	CL (mL/min/kg)	Vss (mL/kg)
D-carnosine	75	5818	108112	9.23	120
L-carnosine	78	4011	89802	11.08	223

## Abbreviations

MCAO	Middle Cerebral Artery Occlusion
TTC	2, 3, 5-Triphenyltetrazolium chloride
AUC	Area under curve
Cmax	Peak serum concentration
ROS	Reactive oxygen species
ICA	Internal carotid artery

## References

[1] Mozaffarian D., Benjamin E.J., Go A.S., Arnett D.K., Blaha M.J., Cushman M., de Ferranti S., Després J.P., Fullerton H.J., Howard V.J. (2015). Heart disease and stroke statistics--2015 update: A report from the American Heart Association. Circulation.

[2] Akpan N., Troy C.M. (2013). Caspase inhibitors: Prospective therapies for stroke. Neuroscientist.

[3] Cheng Y.D., Al-Khoury L., Zivin J.A. (2004). Neuroprotection for ischemic stroke: Two decades of success and failure. NeuroRx.

[4] Chollet F., Rigal J., Marque P., Barbieux-Guillot M., Raposo N., Fabry V., Albucher J.F., Pariente J., Loubinoux I. (2018). Serotonin Selective Reuptake Inhibitors (SSRIs) and Stroke. Curr. Neurol. Neurosci. Rep..

[5] Fisher M. (2011). New approaches to neuroprotective drug development. Stroke.

[6] Hess D.C. (2006). NXY-059: A hopeful sign in the treatment of stroke. Stroke.

[7] Kennedy J., Buchan A.M. (2005). C-EPO: Ready for prime-time preconditioning?. Cerebrovasc. Dis..

[8] Lo E.H., Dalkara T., Moskowitz M.A. (2003). Mechanisms, challenges and opportunities in stroke. Nat. Rev. Neurosci..

[9] Macrae I.M., Allan S.M. (2018). Stroke: The past, present and future. Brain Neurosci. Adv..

[10] Boldyrev A.A., Aldini G., Derave W. (2013). Physiology and pathophysiology of carnosine. Physiol. Rev..

[11] Bae O.N., Serfozo K., Baek S.H., Lee K.Y., Dorrance A., Rumbeiha W., Fitzgerald S.D., Farooq M.U., Naravelta B., Bhatt A. (2013). Safety and efficacy evaluation of carnosine, an endogenous neuroprotective agent for ischemic stroke. Stroke.

[12] Majid A. (2014). Neuroprotection in stroke: Past, present, and future. ISRN Neurol..

[13] Min J., Senut M.C., Rajanikant K., Greenberg E., Bandagi R., Zemke D., Mousa A., Kassab M., Farooq M.U., Gupta R. (2008). Differential neuroprotective effects of carnosine, anserine, and N-acetyl carnosine against permanent focal ischemia. J. Neurosci. Res..

[14] Rajanikant G.K., Zemke D., Senut M.C., Frenkel M.B., Chen A.F., Gupta R., Majid A. (2007). Carnosine is neuroprotective against permanent focal cerebral ischemia in mice. Stroke.

[15] Boldyrev A., Bulygina E., Leinsoo T., Petrushanko I., Tsubone S., Abe H. (2004). Protection of neuronal cells against reactive oxygen species by carnosine and related compounds. Comp. Biochem. Physiol. B Biochem. Mol. Biol..

[16] Fontana M., Pinnen F., Lucente G., Pecci L. (2002). Prevention of peroxynitrite-dependent damage by carnosine and related sulphonamido pseudodipeptides. Cell Mol. Life Sci..

[17] Abe H., Dobson G.P., Hoeger U., Parkhouse W.S. (1985). Role of histidine-related compounds to intracellular buffering in fish skeletal muscle. Am. J. Physiol..

[18] Guiotto A., Calderan A., Ruzza P., Borin G. (2005). Carnosine and carnosine-related antioxidants: A review. Curr. Med. Chem..

[19] Hipkiss A.R. (2009). Carnosine and its possible roles in nutrition and health. Adv. Food. Nutr. Res..

[20] Lai T.W., Zhang S., Wang Y.T. (2014). Excitotoxicity and stroke: Identifying novel targets for neuroprotection. Prog. Neurobiol..

[21] Baek S.H., Noh A.R., Kim K.A., Akram M., Shin Y.J., Kim E.S., Yu S.W., Majid A., Bae O.N. (2014). Modulation of mitochondrial function and autophagy mediates carnosine neuroprotection against ischemic brain damage. Stroke.

[22] Aldini G., Orioli M., Rossoni G., Savi F., Braidotti P., Vistoli G., Yeum K.J., Negrisoli G., Carini M. (2011). The carbonyl scavenger carnosine ameliorates dyslipidaemia and renal function in Zucker obese rats. J. Cell Mol. Med..

[23] Bellia F., Vecchio G., Rizzarelli E. (2012). Carnosine derivatives: New multifunctional drug-like molecules. Amino Acids.

[24] Riedl E., Koeppel H., Brinkkoetter P., Sternik P., Steinbeisser H., Sauerhoefer S., Janssen B., van der Woude F.J., Yard B.A. (2007). A CTG polymorphism in the CNDP1 gene determines the secretion of serum carnosinase in Cos-7 transfected cells. Diabetes.

[25] Janssen B., Hohenadel D., Brinkkoetter P., Peters V., Rind N., Fischer C., Rychlik I., Cerna M., Romzova M., de Heer E. (2005). Carnosine as a protective factor in diabetic nephropathy: Association with a leucine repeat of the carnosinase gene CNDP1. Diabetes.

[26] Vistoli G., Orioli M., Pedretti A., Regazzoni L., Canevotti R., Negrisoli G., Carini M., Aldini G. (2009). Design, synthesis, and evaluation of carnosine derivatives as selective and efficient sequestering agents of cytotoxic reactive carbonyl species. ChemMedChem.

[27] Margolis F.L. (1974). Carnosine in the primary olfactory pathway. Science.

[28] Cararo J.H., Streck E.L., Schuck P.F., Ferreira Gda C. (2015). Carnosine and Related Peptides: Therapeutic Potential in Age-Related Disorders. Aging Dis..

[29] Teufel M., Saudek V., Ledig J.P., Bernhardt A., Boularand S., Carreau A., Cairns N.J., Carter C., Cowley D.J., Duverger D. (2003). Sequence identification and characterization of human carnosinase and a closely related non-specific dipeptidase. J. Biol. Chem..

[30] Unno H., Yamashita T., Ujita S., Okumura N., Otani H., Okumura A., Nagai K., Kusunoki M. (2008). Structural basis for substrate recognition and hydrolysis by mouse carnosinase CN2. J. Biol. Chem..

[31] Otani H., Okumura N., Hashida-Okumura A., Nagai K. (2005). Identification and characterization of a mouse dipeptidase that hydrolyzes L-carnosine. J. Biochem..

[32] Pandya V., Ekka M.K., Dutta R.K., Kumaran S. (2011). Mass spectrometry assay for studying kinetic properties of dipeptidases: Characterization of human and yeast dipeptidases. Anal. Biochem..

[33] Mahmood I. (2014). Naive pooled-data approach for pharmacokinetic studies in pediatrics with a very small sample size. Am. J. Ther..

[34] KuKanich B., Huff D., Riviere J.E., Papich M.G. (2007). Naive averaged, naive pooled, and population pharmacokinetics of orally administered marbofloxacin in juvenile harbor seals. J. Am. Vet. Med. Assoc..

[35] Xie Z., Baba S.P., Sweeney B.R., Barski O.A. (2013). Detoxification of aldehydes by histidine-containing dipeptides: From chemistry to clinical implications. Chem. Biol. Interact..

[36] Anderson E.J., Vistoli G., Katunga L.A., Funai K., Regazzoni L., Monroe T.B., Gilardoni E., Cannizzaro L., Colzani M., De Maddis D. (2018). A carnosine analog mitigates metabolic disorders of obesity by reducing carbonyl stress. J. Clin. Investig..

[37] Caruso G., Fresta C.G., Fidilio A., O′Donnell F., Musso N., Lazzarino G., Grasso M., Amorini A.M., Tascedda F., Bucolo C. (2019). Carnosine Decreases PMA-Induced Oxidative Stress and Inflammation in Murine Macrophages. Antioxidants (Basel).

[38] Peters V., Calabrese V., Forsberg E., Volk N., Fleming T., Baelde H., Weigand T., Thiel C., Trovato A., Scuto M. (2018). Protective Actions of Anserine Under Diabetic Conditions. Int. J. Mol. Sci..

[39] Schön M., Mousa A., Berk M., Chia W.L., Ukropec J., Majid A., Ukropcová B., de Courten B. (2019). The Potential of Carnosine in Brain-Related Disorders: A Comprehensive Review of Current Evidence. Nutrients.

[40] Zhang L., Yao K., Fan Y., He P., Wang X., Hu W., Chen Z. (2012). Carnosine protects brain microvascular endothelial cells against rotenone-induced oxidative stress injury through histamine H1and H2receptors in vitro. Clin. Exp. Pharmacol. Physiol..

[41] Khama-Murad A., Mokrushin A., Pavlinova L. (2011). Neuroprotective properties of l-carnosine in the brain slices exposed to autoblood in the hemorrhagic stroke model in vitro. Regul. Pept..

[42] Bae O.N., Majid A. (2013). Role of histidine/histamine in carnosine-induced neuroprotection during ischemic brain damage. Brain Res..

[43] Orioli M., Aldini G., Beretta G., Facino R.M., Carini M. (2005). LC-ESI-MS/MS determination of 4-hydroxy-trans-2-nonenal Michael adducts with cysteine and histidine-containing peptides as early markers of oxidative stress in excitable tissues. J. Chromatogr. B Analyt. Technol. Biomed. Life Sci..

[44] Peiretti P.G., Medana C., Visentin S., Giancotti V., Zunino V., Meineri G. (2011). Determination of carnosine, anserine, homocarnosine, pentosidine and thiobarbituric acid reactive substances contents in meat from different animal species. Food. Chem..

[45] Berridge M.V., Herst P.M., Tan A.S. (2005). Tetrazolium dyes as tools in cell biology: New insights into their cellular reduction. Biotechnol. Annu. Rev..

[46] Hasan M.M., Islam M.S., Hoque K.M.F., Haque A., Reza M.A. (2019). Effect of Citrus macroptera Fruit Pulp Juice on Alteration of Caspase Pathway Rendering Anti-Proliferative Activity against Ehrlich’s Ascites Carcinoma in Mice. Toxicol. Res..

